# Spontaneous Rhinorrhea mimicking sinusitis

**DOI:** 10.11604/pamj.2015.20.97.5748

**Published:** 2015-02-04

**Authors:** Selcuk Ozdogan, Yusuf Emrah Gergin, Sinem Gergin, Necati Tatarli, Tufan Hicdonmez

**Affiliations:** 1 Lutfi Kirdar Kartal Training and Research Hospital, Istanbul, Turkey; 2 Marmara University, Department of Anatomy, Istanbul, Turkey

**Keywords:** Rhinorrhea, spontaneous rhinorrhea, sinusitis

## Abstract

Spotaneous or non-traumatic cerebrospinal fluid rhinorrhea is an uncommon condition and may present a diagnostic challenge to clinicians. This condition is often being misdiagnosed for allergic rhintis or chronic sinusitis since the precipitating cause is not readily apperent in most patients. The mechanism of rhinorrhea is stil not completely clarified. We describe a case of this condition occuring in association with allergic rhinitis and sinusitis. A 52 year-old, obese female patient presented with two weeks history of bilateral clear nasal discharge and postural headache. Sample of nasal discharge tested for glucose and protein. The result was that the collection fluid was cerebrospinal fluid. The origin of cerebrospinal fluid fistula could not be identified despite the diagnostic tests.

## Introduction

Non-traumatic and non-surgical spontaneous cerebrospinal fluid (CSF) rhinorrhea constitutes only 3-4% of all CSF rhinorrhea [1–6]. Spotaneous or non-traumatic CSF rhinorrhea may present a diagnostic challenge to clinicians. When there is a fistula between dura and the skull base, CSF rhinorrhea occurs and can be divided into two major groups such as traumatic and non-traumatic rhinorrhea [7, 8]. Non-traumatic CSF rhinorrhea is seen in middle aged group like older than 30 years old. It is an insidious disease and it can be confused with allergic rhinitis. Unlike the more common traumatic or post-surgical leaks, pneumocephalus and smell impairment is rare. The non-traumatic group is associated with brain tumors which are known to erode skull like cholestatoma or tuberculoma, skull base congenital defects and meningoceles or meningoencephalocele. Approximately 80% of all cases of CSF rhinorrhea are caused by head trauma, while another 16% result from surgery of skull base [4]. Rhinitis induced spontaneous CSF rhinorrhea is extremely rare [3, 4, 6, 9]. A rare case of this condition in association with allergic rhinitis is documanted.

## Patient and observation

**Figure 1:**
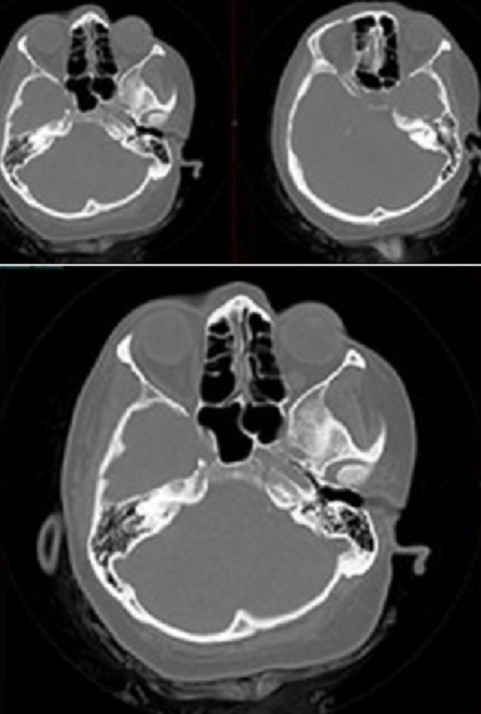
Axial images of paranasal computed tomography that shows no bone defect for rhinorrhea

A 52 year-old obese female patient peresented into emergency room with one week history of bilateral clear watery discharge and postural headache. The history revealed that she had been previously evaluated and treated for rhinosinusitis with antibiotic theraphy and nasal steroids by otorhinolaryngology. She had no history head trauma. Patients systemic physical examination was normal. On her neurologic examination there was not any abnormal findings including sense of smell. A sample of rhinorrhea was collected for glucose and protein examination and the results were 95mg/dl glucose and 43.8 mg/dl protein. CSF rhinorrhea was considered as the primary diagnosis. Non-contrast paranasal CT result was normal (Figure 1). But she has reservoir sign. Continious lumbar drainage was implanted for six days. After the 3rd day of drainage, CSF rhinorrhea discharge stopped. She made a good recover and in follow up examination, she has no rhinorrhea.

## Discussion

About 80-85% cases of CSF rhinorrhea is caused after head trauma and 16% result from paranasal or skull base surgery [1, 5, 7]. Approximately 3-4% of all cases are spontaneous [3, 4, 7]. Rhinorrhea was subdivided into two categories by O'Connell. He describe primary rhinorrhea is seen without a reason and secondary rhinorrhea diagnosed with a cause [7]. In secondary rhinorrhea group, hydocephalus and tumors are the most common causes. But Ommaya described another classification. He subdivided spontaneous CSF rhinorrhea into two categories: high pressure, caused by hydocephalus and tumors; low pressure, caused by osteomyelitis, focal atrophy, congenital anomalies [7]. Also rhinorrhea can be a symptom of common conditions involving nasopharynx, such as rhinosinusitis [5, 8]. Rhinosinusitis is usually associated by nasal discharge, paranasal discharge, congestion, headache and fever. Rhinorrhea also occurs with CSF leaks. In this situation it is worsen by valsalva maneuver, bending forward and it is usually unilateral. Corticosteroids and antihistaminics are useless in CSF rhinorrhea. It is unilateral and also it may not always correlate with the fistula location [6, 10].

Fluid material should be collected for testing Beta2-transferrin immunofixation when there is a clinical suspicion of CSF leakage. Accompanied by this cranial CT and cranial MRI should be performed for determination of the location of defect. In our case cranial CT was reported without abnormalities. She is also diagnosed by detecting glucose and protein levels of nasal discharge with a collection of sample during the workup.

## Conclusion

Spontaneous CSF rhinorrhea is a condition that mimics rhinosinusitis and allergic rhinitis. It may be taken into consideration and is critical that pyhsicians include this condition in differential diagnosis.
